# Non-Invasive Distal Airway Proteomics by Particles in Exhaled Air (PExA) Sampling Demonstrates Shared Proteomic Features with Matched Bronchoalveolar Lavage

**DOI:** 10.3390/biom16101396

**Published:** 2026-09-24

**Authors:** Kiara M. Knuckey, Sjane Timmins, Amy Pham, Maxine E. Tan, Penny L. Groves, Viviana P. Lutzky, Subash K. Rai, Alan M. Carew, Daniel C. Chambers, Simon H. Apte

**Affiliations:** 1 Queensland Lung Transplant Service, The Prince Charles Hospital, Brisbane, QLD 4032, Australia; k.knuckey@uq.edu.au (K.M.K.);; 2 School of Medicine, Faculty of Health, Medicine and Behavioural Sciences, The University of Queensland, Brisbane, QLD 4072, Australia; 3 Australian Centre for Cellular Ageing, The Prince Charles Hospital, Brisbane, QLD 4032, Australia

**Keywords:** exhaled breath, PExA, proteomics, silicosis, bronchoalveolar lavage

## Abstract

Repeated molecular sampling of the distal airways is constrained by the invasive nature of bronchoalveolar lavage (BAL), limiting its use in healthy control populations, longitudinal studies, clinical trials and occupational screening. Particles in exhaled air (PExA) sampling offers a non-invasive approach to distal-airway sampling, although whether it captures the same proteins and relative protein abundance as BAL has not been established. We performed the first proof-of-principle high-dimensional proteomic comparison of matched BAL and PExA samples collected from three individuals with silicosis using the SomaScan 11K platform. Within individuals, PExA and BAL shared a substantial subset of detectable proteins and showed similar cross-protein signal patterns, with Pearson correlation coefficients ranging from 0.48 to 0.71. Analytes recurrently detected across all three PExA samples were strongly enriched among those consistently detected in BAL and included pulmonary surfactant proteins and mediators associated with epithelial injury, alveolar macrophage function, inflammation and fibrosis. The PExA findings also showed substantial overlap with an independent published PExA-only proteomic dataset. These proof-of-principle findings show that non-invasive PExA sampling can capture a biologically meaningful subset of the matched BAL proteome. PExA may therefore offer a practical approach for repeated molecular profiling of the distal airways and warrants further evaluation for respiratory biomarker discovery and disease monitoring.

## 1. Introduction

Molecular sampling of the distal airways is important for understanding respiratory diseases in which clinically relevant pathology occurs within them. Bronchoalveolar lavage (BAL) remains the reference method for obtaining biological material from this compartment and supports cellular, microbiological and molecular analyses [1]. However, BAL requires bronchoscopy, local anaesthesia or sedation, and instillation of saline into a selected lung segment. Its invasive nature limits repeated sampling, restricts recruitment of healthy control populations and presents a substantial barrier to longitudinal studies, clinical trials and occupational screening. BAL measurements are also affected by variable dilution with instilled fluid and reflect a selected lung region rather than necessarily providing an integrated assessment of distal-airway biology.

Particles in exhaled air (PExA) sampling provides a non-invasive approach to recovering non-volatile material from respiratory tract lining fluid. The seminal PExA methodology combined optical particle counting with inertial impaction, allowing exhaled particles to be counted, sized and collected for subsequent molecular analysis [2]. Particle generation is promoted by closure and reopening of small airways, during which fluid films rupture and release aerosolized droplets. A standardised manoeuvre involving exhalation to residual volume, breath holding, inhalation to total lung capacity and controlled exhalation is now commonly used to increase and standardise particle recovery [3]. Because the number and size of collected particles are measured, molecular signals can be related to the estimated mass of recovered material. This distinguishes PExA from exhaled breath condensate, in which respiratory material is highly diluted by condensed water vapour [4].

Early mass-spectrometric analysis of exhaled particles identified surfactant-associated phospholipids, supporting their origin from respiratory tract lining fluid [2]. Bredberg et al. [5] subsequently performed the first mass-spectrometric proteomic analysis of PExA and demonstrated that collected particles contained surfactant proteins, secreted proteins and other proteins associated with respiratory tract lining fluid. Later studies using mass spectrometry, aptamer-based assays and proximity-extension platforms showed that PExA can recover broader protein profiles and identify molecular differences associated with asthma, smoking, chronic cough, lung adenocarcinoma, coronavirus disease and chronic lung allograft dysfunction [6,7,8,9,10,11]. Collectively, these studies showed that PExA can recover reproducible, biologically plausible protein profiles and detect disease-associated differences across several respiratory conditions. However, none included matched BAL from the same participants. Comparisons with proteins reported in separate BAL studies could therefore demonstrate overlap in protein identity, but not within-person correlations in protein detection or relative abundance between the two matrices. Bredberg et al. [5], for example, identified 124 proteins in pooled PExA samples, of which 103 (83%) had previously been reported in independent published BAL proteomic data; however, BAL was not collected from the participants contributing the PExA samples.

Direct human comparisons with invasive airway samples have remained limited to selected molecular components. Behndig et al. [12] measured albumin and surfactant protein A in matched PExA, BAL and bronchial wash samples from 15 healthy participants. PExA albumin and albumin-corrected surfactant protein A were associated with BAL but not bronchial wash, findings consistent with a predominantly distal-airway origin for PExA material; however, only two prespecified proteins were examined. In a controlled endotoxin-challenge study, increases in interleukin-6 and interleukin-8 were detected in PExA alongside inflammatory responses in BAL after segmental challenge and induced sputum after inhaled challenge. Analysis of the same cohort also showed that PExA phospholipid composition was more closely related to BAL than to induced sputum [13,14]. The only previous high-dimensional proteomic comparison of exhaled particles and BAL was performed in a porcine lung-transplant model, and although Niroomand et al. [15] also analysed exhaled particles from human lung-transplant recipients, matched BAL proteomics was not performed in the human cohort.

Thus, previous work has shown that PExA contains lung-derived proteins, can detect disease-associated molecular profiles and shares selected biochemical features with BAL. What has not been determined is whether the human PExA proteome correlates with the matched BAL proteome within the same individual across hundreds of protein analytes. This distinction is important if PExA is to be used for repeated molecular profiling, biomarker discovery or disease monitoring. We therefore performed the first proof-of-principle high-dimensional proteomic comparison of matched human BAL and PExA samples using the SomaScan 11K platform in individuals with silicosis. Silicosis is a debilitating occupational lung disease caused by inhalation of respirable crystalline silica, with a marked increase in incidence in Australia over the past decade, particularly among workers exposed during engineered-stone processing [16]. These participants were recruited through an ongoing silicosis research programme at our hospital that enabled collection of matched BAL and PExA samples [17,18]. We examined whether protein abundance patterns in PExA correlated with matched BAL, whether analytes recurrently detected in PExA were enriched among those recurrently detected in BAL, and whether shared and matrix-associated analyte compartments represented biologically coherent features of distal-airway biology.

## 2. Materials and Methods

### 2.1. Study Population

Samples were obtained from three patients with silicosis undergoing clinically indicated bronchoscopy at The Prince Charles Hospital. Participants provided a PExA sample either on the day of bronchoscopy or up to 24 h beforehand. All participants provided written informed consent under a protocol approved by The Prince Charles Hospital Human Research Ethics Committee (HREC/2018/QPCH/44293).

### 2.2. Bronchoalveolar Lavage

BAL was performed during clinically indicated bronchoscopy according to international guidelines [19]. Sterile normal saline (120 mL total) was instilled into the anterior segment of the right upper lobe and recovered using gentle wall suction without dwell time. Approximately 20 mL of recovered fluid per patient was centrifuged at 800× *g* for 10 min, with supernatant removed and stored in 1 mL aliquots at −80 °C.

BAL protein concentrations were quantified using a NanoDrop One spectrophotometer (Thermo Fisher Scientific, Scoresby, VIC, Australia). BAL samples were diluted to 200 μg/mL using sterile PBS and 112 μL was aliquoted for SomaScan analysis. All samples were stored at −80 °C prior to shipment on dry ice.

### 2.3. PExA

Matched PExA samples were collected using the PExA 2.1 platform (PExA AB, Gothenburg, Sweden). Participants performed a standardised breathing manoeuvre consisting of repeated exhalations to residual volume followed by breath hold for 5 s, rapid inhalation and controlled exhalation back to residual volume to promote distal airway closure and reopening events associated with particle generation. True blank controls (*n* = 3) were generated using a 3 L spirometer calibrator in place of a participant, with 15 breathing-equivalent manoeuvres performed per control.

Collected particles were inertially impacted onto dried blood spot (DBS) membranes for processing. PExA samples were eluted from DBS membranes using SB17T buffer (SomaLogic, Boulder, CO, USA) containing 0.4% Tween-20 and protease inhibitor cocktail (Thermo Scientific #87786). Elution volumes were adjusted according to collected PExA mass across samples to achieve a uniform final particle concentration, with a minimum elution volume of 128 µL, consistent with previously published PExA SomaScan workflows [8]. True blank controls were eluted in 128 µL of SB17T regardless of collected mass. Samples were incubated in a shaking water bath for 30 min (30 °C, 100 rpm) and centrifuged at 10,000× *g* for 2 min, and 112 μL of supernatant was aliquoted for SomaScan analysis and frozen at −80 °C prior to shipment on dry ice.

### 2.4. SomaScan Data Analysis

Proteomic analysis was performed using the SomaScan 11K platform (SomaLogic, Boulder, CO, USA). Samples were analysed on separate plates to permit maximal analyte detection given the low particle concentration in PExA compared to BAL. SomaLogic-normalised medNormSMP ADAT files were analysed, in which sample-level normalisation had been applied independently within each study by SomaLogic prior to downstream analysis. SomaScan analytes were considered detectable when signal exceeded the mean plus 3 standard deviations of true blank PExA controls for PExA samples and assay buffer controls for BAL samples. A recurrent PExA detection set was defined as analytes detected in all three PExA samples, analogous to prior PExA SomaScan studies that used detection above the limit of detection (LOD) in all samples as the most stringent analysis set [8]. Because the SomaScan 11K platform includes multiple analytes recognising different epitopes or isoforms of the same protein target, descriptive detection, correlation and Fisher enrichment analyses were performed at the SomaScan analyte/SeqID level after exclusion of internal-use, non-human and assay-control targets. Pearson and Spearman correlations were calculated using log2-transformed signal intensities for analytes detected in both BAL and PExA for each matched subject. Enrichment of analytes detected in all three PExA samples among those detected in all three BAL samples was assessed using a two-sided Fisher’s exact test against a background of 10,751 reportable human SomaScan analytes (Data S1). To assess the robustness of the BAL–PExA comparisons to analytes close to the detection threshold, a sensitivity analysis was performed in which correlations and recurrent-detection enrichment were recalculated after requiring analyte signal to exceed 1.5-fold or 2-fold the matrix-specific detection threshold in both BAL and PExA (Data S4 and S5, respectively). These more stringent cut-offs were used as sensitivity filters and did not replace the primary detection criterion. For pathway interpretation, recurrent BAL-exclusive, shared BAL–PExA and PExA-exclusive analyte sets were mapped by SeqID to the UniProt accessions provided in the SomaScan 11K annotation. Annotations containing multiple UniProt accessions were expanded, and duplicate UniProt identifiers were removed. The resulting compartment-specific protein lists were submitted separately to the STRING web interface using *Homo sapiens*, query proteins only with no additional interactors, and the whole-genome statistical background. Functional enrichments with a false discovery rate ≤0.05 were considered significant (Data S2) [20]. Analyses were performed in Microsoft Excel for Mac (version 16.110.2) and plots were generated in RStudio (version 2026.01.0 + 392). For exploratory cellular-context interpretation, the 12 recurrent PExA-exclusive genes were manually queried in the Kaminski/Rosas dataset within the idiopathic pulmonary fibrosis (IPF) Cell Atlas [21].

## 3. Results

### 3.1. Study Cohort

Matched BAL and PExA samples were collected from three individuals with silicosis recruited through The Prince Charles Hospital, Brisbane, Australia [17]. Subject 1 was a 42-year-old male ex-smoker with simple silicosis following 15 years of occupational silica exposure. Lung function testing demonstrated forced expiratory volume in 1 s (FEV1) 110.5% predicted, forced vital capacity (FVC) 108.2% predicted and diffusing capacity of the lungs for carbon monoxide (DLCO) 76.9% predicted. Subject 2 was a 60-year-old male non-smoker with simple silicosis following 17 years of occupational silica exposure with FEV1 95.6% predicted, FVC 100.5% predicted and DLCO 78.6% predicted. Subject 3 was a 54-year-old male current smoker with simple silicosis following 30 years of occupational silica exposure with FEV1 77.3% predicted, FVC 69.2% predicted and DLCO 45.4% predicted. PExA samples were collected at 3, 24 and 24 h before bronchoscopy, yielding 106 ng, 215.7 ng and 210.9 ng of material over 11, 3 and 6 breathing manoeuvres for Subjects 1, 2 and 3, respectively. Total accumulated particle counts were 241,897, 456,932 and 390,139 for Subjects 1–3, respectively. Particle-size distributions were similar across participants, with relative particle counts peaking at a characteristic diameter of approximately 0.83 μm and the greatest relative particle mass occurring at approximately 0.83–0.99 μm (Figure S1). PExA samples were eluted in 128 μL, 260.5 μL, and 254.7 μL for Subjects 1, 2 and 3 respectively, to achieve a uniform final concentration of 0.828 ng/μL across samples.

### 3.2. Correlations Between PExA and BAL

Matched BAL and PExA proteomes demonstrated positive subject-specific correlations across all analysed individuals. Subject 2 demonstrated the strongest correlation between BAL and PExA signal abundance (Pearson r = 0.71, Spearman ρ = 0.75, *n* = 688 analytes) (Figure 1B; Data S1). Positive correlations were also observed in Subject 1 (Pearson r = 0.55, Spearman ρ = 0.62, *n* = 343 analytes) (Figure 1A; Data S1) and Subject 3 (Pearson r = 0.48, Spearman ρ = 0.49, *n* = 440 analytes) (Figure 1C; Data S1); all Pearson and Spearman correlations were significant at *p* < 0.001. Of 162 analytes detected in all three PExA samples, 131 were also detected in all three BAL samples, compared with 32.73 expected by chance. Recurrently detected PExA analytes were strongly enriched among recurrently detected BAL analytes (4.00-fold observed-to-expected enrichment; two-sided Fisher’s exact test OR = 17.70, *p* = 1.02 × 10^−62^) (Data S1). The findings were robust to progressively more stringent detection criteria. When analytes were required to exceed 1.5-fold the matrix-specific detection threshold in both BAL and PExA, 108, 55 and 67 co-detected analytes remained for Subjects 1–3, respectively, with Pearson correlations of r = 0.53, 0.59 and 0.34. At a 2-fold threshold, 79, 34 and 41 analytes remained, with Pearson correlations of r = 0.51, 0.61 and 0.34, respectively. Recurrent PExA detections also remained strongly enriched among recurrent BAL detections at both stricter thresholds (1.5-fold: 32/37 recurrent PExA analytes shared with BAL; 2-fold: 16/21 shared with BAL). Full sensitivity analyses are provided in Datas S4 and S5.

### 3.3. Analyte Pathway Interpretations

SomaScan analytes were classified into three biologically interpretable compartments (Figure 2; Data S2). BAL-exclusive analytes ranged from 2282 to 4669 per subject, with 1464 analytes recurrently detected in all three BAL samples and absent from all matched PExA samples. These analytes mapped to 1425 unique UniProt identifiers. The mapped BAL-exclusive protein set was enriched for metabolic and catabolic pathways and included ABCG1, ACO2, ACP5 and CTSH.

Analytes detected in both matched BAL and PExA samples ranged from 343 to 688 per subject, with 131 recurrently co-detected across all three individuals. These analytes mapped to 129 unique UniProt identifiers and included C5, C6, C9, SERPING1, CFH, CFI, SFTPD, CHI3L1, MFGE8 and KLKB1. STRING analysis identified enrichment for extracellular innate immune and airway lining-fluid processes, including complement and coagulation pathways and humoral immune responses.

PExA-exclusive analytes ranged from 92 to 415 per subject, with 12 recurrently detected across all three individuals and absent from matched BAL samples. These mapped to 12 unique UniProt identifiers and included P2RY12, RGS3, SNX7 and CDKL5. No significant functional enrichment was detected for this small protein set. Exploratory review of the Kaminski/Rosas dataset within the IPF Cell Atlas showed that the corresponding genes were expressed across several epithelial, immune, stromal and vascular cell populations [21].

### 3.4. Comparison with a Previously Published PExA Proteome

We compared the analytes detected in the current PExA samples with the 207 analytes reported in the SomaScan 1.3K PExA study by Östling et al. [8]. Among these, 56 were recurrently detected in all three PExA samples in the current study and 126 were detected in at least one PExA sample. Four previously reported analytes were not represented on the SomaScan 11K platform. Overall, 1614 reportable human analytes were detected in at least one PExA sample in the current study (Data S3).

## 4. Discussion

This study provides the first proof-of-principle high-dimensional proteomic comparison of matched human BAL and PExA samples. Three principal findings emerged. First, PExA and BAL showed consistent positive within-participant correlations in relative analyte abundance, with Pearson correlations ranging from 0.48 to 0.71 and Spearman correlations from 0.49 to 0.75 across 343–688 co-detected analytes per participant. Second, 131 of the 162 analytes recurrently detected in PExA were also recurrently detected in BAL, representing strong enrichment beyond that expected by chance. Third, the recurrent shared analytes formed a biologically coherent extracellular compartment containing surfactant-associated, complement, coagulation and innate immune proteins. Importantly, the positive within-participant correlations and enrichment of recurrent PExA detections within BAL persisted after progressively excluding analytes close to the detection threshold, indicating that the principal findings were not dependent solely on borderline detections. Together, these findings extend previous evidence that PExA contains lung-derived material by demonstrating shared protein detection and correlated relative abundance with BAL within the same individuals.

The earliest proteomic evidence for the pulmonary origin of exhaled particles was provided by Bredberg et al. [5], who identified 124 proteins by liquid chromatography–mass spectrometry in pooled PExA samples from healthy volunteers. Of these, 103 had previously been reported in independent BAL proteomic data, and the identified proteins included surfactant proteins A, B and C, club cell secretory protein, complement proteins and other extracellular proteins. That study established that PExA contained a plausible respiratory tract lining-fluid proteome, but the BAL comparison was made against published protein lists rather than samples obtained from the same participants, and relative abundance was estimated in pooled PExA material. Our findings provide the missing direct human comparison: not only were many proteins present in both matrices, but relative analyte signals were correlated across hundreds of proteins within each matched individual.

Subsequent human proteomic studies showed that PExA could identify protein profiles associated with smoking, asthma and small-airway dysfunction, chronic cough, lung cancer and respiratory infection [6,7,8,9,10]. These studies addressed whether PExA could detect biologically and clinically relevant differences between groups, whereas the present study addressed whether PExA measurements reflect the proteome of matched invasively obtained distal-airway samples. The overlap with the independent SomaScan 1.3K PExA dataset reported by Östling et al. [8] provides external support for the biological plausibility of the detected PExA proteome despite differences in cohort composition and assay generation. However, the greater number of analytes detected in the current study should not be interpreted solely as improved biological recovery, because platform coverage, cohort composition, sample mass and detection definitions differed between studies.

Our results also extend previous matched biochemical comparisons. Behndig et al. [12] demonstrated that PExA albumin and albumin-corrected surfactant protein A were associated with BAL but not bronchial wash, providing targeted evidence that PExA preferentially reflects distal rather than proximal airway material. Holz et al. [13] showed that selected inflammatory cytokines increased in PExA following experimental endotoxin challenge alongside inflammatory responses in BAL or induced sputum, while Larsson et al. [14] found that PExA phospholipid composition was more closely related to BAL than to induced sputum. These studies demonstrated related patterns for individual proteins, inflammatory responses or lipids. The current study expands this evidence from a small number of prespecified measurements to hundreds of simultaneously measured protein analytes. Niroomand et al. [15] similarly reported proteomic overlap between exhaled particles and BAL in a porcine lung-transplant model, but matched proteomics was not performed in their human cohort. Our results therefore translate this high-dimensional comparison to matched human samples.

The composition of the shared and matrix-associated analyte compartments provides further insight into what each sampling method recovers. Recurrently shared proteins included SFTPD, CHI3L1, MFGE8, complement components and regulators, and coagulation-associated proteins. Their enrichment for extracellular innate immune and airway lining-fluid pathways is consistent with the proposed origin of PExA particles from fluid films lining the distal airways. In human IPF, terminal complement components C5–C9 have been reported to be increased in BAL, supporting the relevance of terminal complement activation to fibrotic lung disease [22]. Some complement- and coagulation-associated signals in BAL may also reflect minor blood contamination during bronchoscopy and BAL collection. Experimental studies in murine bleomycin models have implicated surfactant protein D (SP-D) in alveolar macrophage regulation and fibrotic lung remodelling [23], while a systematic review and meta-analysis of human interstitial lung disease (ILD) studies found circulating SP-D to be associated with ILD occurrence and clinical outcomes [24]. CHI3L1 has been linked to epithelial injury, alternative macrophage activation and fibroproliferative responses in studies combining observations in human IPF with experimental murine models [25]. More recent in vitro and murine studies have further implicated CHI3L1 in profibrotic macrophage–fibroblast signalling [26].

The biological interpretation of these shared proteins should also be considered in the context of the study population. Because all participants had silicosis and there was no healthy-control comparison, their detection should not be interpreted as a silicosis-specific signature. Silicosis itself may also influence the composition of both sampled matrices. Human BAL studies in silicosis have demonstrated altered alveolar macrophage phenotypes, increased BAL fluid ferritin, substantial changes in BAL-cell gene expression and alterations in the BAL fluid metabolome, providing direct evidence that the molecular composition of the alveolar compartment is modified by disease [27]. Retained crystalline silica, alveolar macrophage accumulation and activation, epithelial injury and fibrotic remodelling may alter the proteins present in distal-airway lining fluid and consequently those recovered by BAL or PExA. Accordingly, the present findings demonstrate matched BAL–PExA relationships within silicosis but should not be assumed to represent the proteomic composition of healthy lungs or other respiratory diseases.

BAL-exclusive analytes were substantially more numerous and were enriched for metabolic and catabolic functions. This likely reflects, at least in part, the much greater biomass recovered by lavage and the presence of intracellular or cell-associated material released from airway and immune cells. BAL also samples fluid from the lavaged airway segment and may recover material from anatomical surfaces and cell populations not efficiently represented in exhaled particles. Conversely, the small recurrent PExA-exclusive compartment may reflect material generated across peripheral lung regions not sampled by the segmental BAL, preferential recovery of airway-interface proteins, or analytical differences between the two matrices. Matrix-specific background, extraction efficiency and detection thresholds may also cause an analyte to be classified as exclusive despite being present below the threshold in the other matrix. These compartments should therefore be described as exclusive under the applied detection criteria, rather than as proteins biologically present in only one compartment.

Perfect agreement between BAL and PExA would not be expected. BAL was obtained from the anterior segment of the right upper lobe, whereas PExA particles arise from repeated airway closure and reopening across multiple peripheral lung regions. BAL proteins are variably diluted by instilled saline, while PExA material is collected by membrane impaction and extracted into a defined buffer volume relative to recovered particle mass. The resulting BAL and PExA preparations may also differ in cellular contribution, protein recovery, aptamer accessibility and matrix-specific background. Because SomaScan relative fluorescence units are normalised assay signals rather than absolute protein concentrations, matrix-specific detection thresholds were required. The present study should therefore be interpreted as proof-of-principle evidence that PExA and matched BAL share a subset of detectable proteins and exhibit similar cross-protein signal patterns, rather than as evidence of quantitative equivalence between the two matrices.

Several limitations should be acknowledged. The study included only three participants, all of whom were men with silicosis, and therefore cannot establish population-level reproducibility, disease specificity or the effect of sex, smoking or disease severity. Subject 3 was a current smoker with lower FVC and DLCO than the other participants, which may have contributed to the lower BAL–PExA correlation observed in this individual; however, this cannot be distinguished from other interindividual differences in a cohort of this size. The statistical significance of each correlation arose from the large number of analytes measured within an individual; the participant, rather than the analyte, remains the relevant unit for generalisation to a wider population. PExA samples were collected between 3 and 24 h before BAL, and the number of breathing manoeuvres required to obtain sufficient material varied between participants. Although the standardised breathing manoeuvre improves particle recovery, substantial interindividual variability in particle generation remains even in healthy populations [3]. However, PExA elution volumes were adjusted according to recovered particle mass to achieve a uniform final particle concentration, such that the number of manoeuvres was not directly equivalent to the material concentration used for SomaScan analysis.

The use of matrix-specific blank controls was necessary but may have influenced classification near the detection threshold. The SomaScan platform has undergone extensive technical evaluation, although most studies have been performed in plasma or serum rather than BAL or PExA. An independent evaluation of the 7K assay using inter-plate technical duplicates from 102 donors demonstrated low analytical variability and examined signal-to-background performance and sources of technical variation [28]. More recently, evaluation of the 11K assay demonstrated median intra-plate and inter-plate coefficients of variation of approximately 3% and 5%, respectively, after normalisation, although inter-batch variability was higher at approximately 8% [29]. Earlier SomaScan platforms have also been evaluated using complementary proteomic approaches. Cross-platform comparison with Olink demonstrated substantial target-dependent variation in agreement between assays, with better agreement for protein targets that had previously undergone orthogonal validation by mass spectrometry or immunoassay [30]. Earlier studies have also reported direct mass-spectrometric assessment of SOMAmer target specificity [31]. The SomaScan platform can contain more than one analyte recognising different epitopes or isoforms of the same protein, and aptamer signal does not provide direct confirmation of protein identity or absolute concentration. No orthogonal immunoassay or mass-spectrometric validation was undertaken in the present study, and the compartment and pathway analyses should therefore be considered exploratory. Future studies should include targeted orthogonal validation of selected PExA proteins, including mass-spectrometric proteomic analysis, together with participants undergoing clinically indicated BAL across different respiratory diseases, independent PExA-only healthy control cohorts and repeated PExA sampling.

These limitations notwithstanding, the findings provide a substantive advance over previous work. PExA should not be regarded as a replacement for BAL when cellular analysis, microbiological assessment or regionally directed sampling is required. Rather, its potential advantage is as a complementary, non-invasive method for repeated molecular assessment when serial bronchoscopy is impractical or unjustified. The demonstration that PExA captures a strongly enriched, biologically coherent subset of the matched BAL proteome supports further evaluation of the method for longitudinal disease monitoring, clinical trials, occupational surveillance and distal-airway biomarker development.

## 5. Conclusions

To our knowledge, this is the first high-dimensional proteomic comparison of matched human BAL and PExA samples and it provides proof-of-principle evidence that PExA and BAL share a subset of detectable proteins and show similar cross-protein signal patterns. PExA captured a recurrent extracellular distal-airway protein signature that showed moderate-to-strong within-person correlations with BAL and was strongly enriched for analytes recurrently detected in BAL. These findings establish that PExA recovers more than isolated abundant surfactant proteins and instead represents a biologically coherent subset of the BAL proteome. Larger studies are required to establish reproducibility across populations and diseases, but PExA shows promise as a complementary platform for repeated and longitudinal molecular assessment of the distal airways.

## Figures and Tables

**Figure 1:**
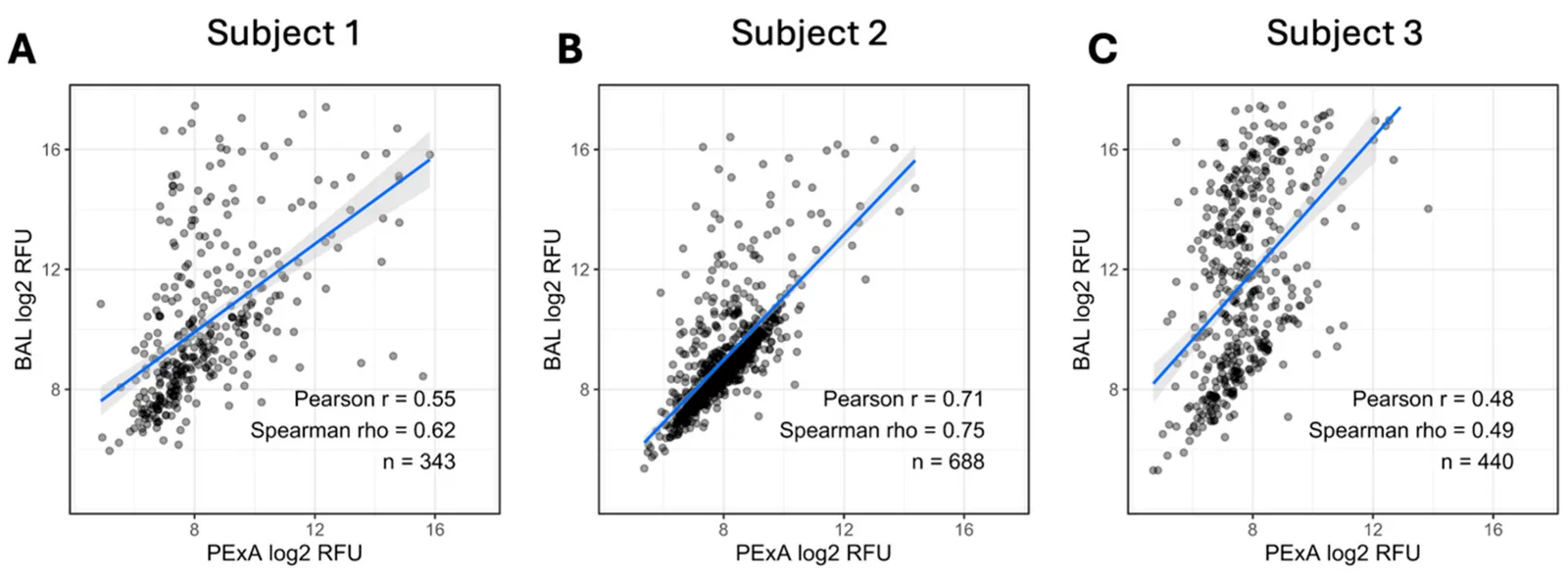
Correlation of log2-normalised SomaScan 11K analyte signals between matched BAL and PExA samples for Subject 1 (**A**), Subject 2 (**B**) and Subject 3 (**C**). Pearson and Spearman coefficients and the number of co-detected analytes (*n*) are shown.

**Figure 2:**
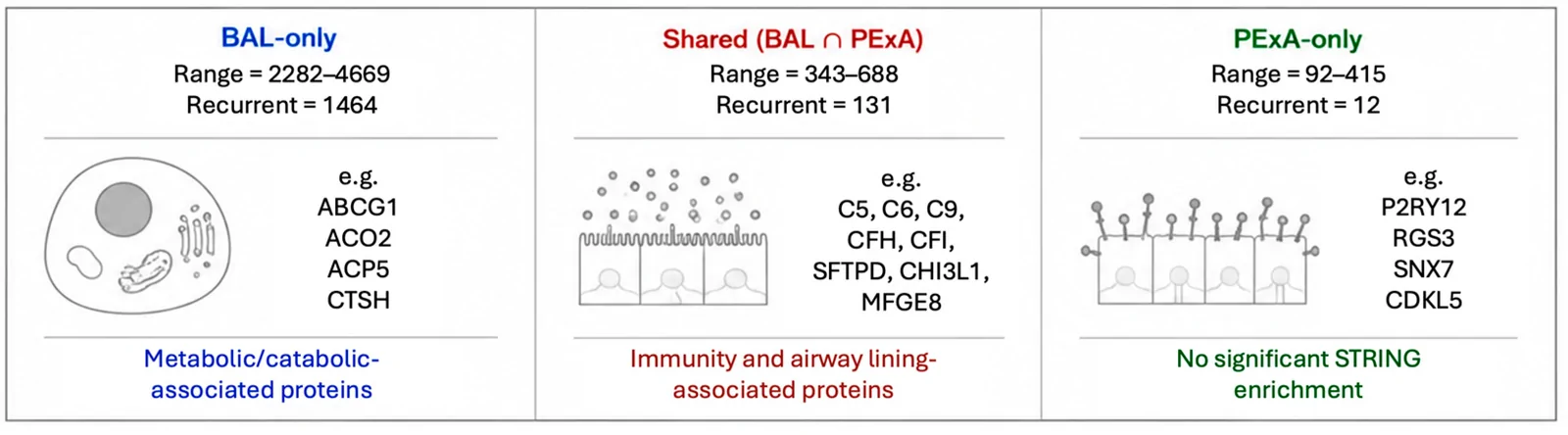
Schematic overview of BAL-only, shared BAL/PExA and PExA-only analyte compartments, together with representative proteins and associated biological pathway enrichments. Ranges of detected analytes across individuals are provided for each compartment. Recurrent shared analytes were detected in both BAL and PExA in all three individuals. Recurrent BAL-exclusive and PExA-exclusive analytes were detected in all three samples of the indicated matrix and absent from all three samples of the other matrix.

## Data Availability

The original contributions presented in this study are included in the article and Supplementary Materials. Further inquiries can be directed to the corresponding author.

## Supplementary Materials

The following supporting information can be downloaded at https://www.mdpi.com/article/10.3390/biom16101396/s1: Figure S1: Particle-size distributions of PExA samples; Data S1: SomaScan calculations and outputs; Data S2: STRING input lists and enrichment outputs; Data S3: Detected PExA analytes compared to Östling; Data S4: 1.5-fold detection-threshold sensitivity analysis; Data S5: 2-fold detection-threshold sensitivity analysis.

## Abbreviations

The following abbreviations are used in this manuscript:

BAL	Bronchoalveolar lavage
PExA	Particles in exhaled air
DBS	Dried blood spot
LOD	Limit of detection
IPF	Idiopathic pulmonary fibrosis
FEV1	Forced expiratory volume in 1 s
FVC	Forced vital capacity
DLCO	Diffusing capacity of the lungs for carbon monoxide
SP-D	Surfactant protein D
ILD	Interstitial lung disease
