# Supplementary material for: Non-Invasive Distal Airway Proteomics by Particles in Exhaled Air (PExA) Sampling Demonstrates Shared Proteomic Features with Matched Bronchoalveolar Lavage
Source: Biomolecules. 2026 Sep 24;16(10):1396. doi: 10.3390/biom16101396 (PMC13702294; doi:10.3390/biom16101396)
Supplement: Supplementary file 1 [file biomolecules-16-01396-s001.zip › biomolecules-4522712-final-Supplementary files/Figure S1.pdf]

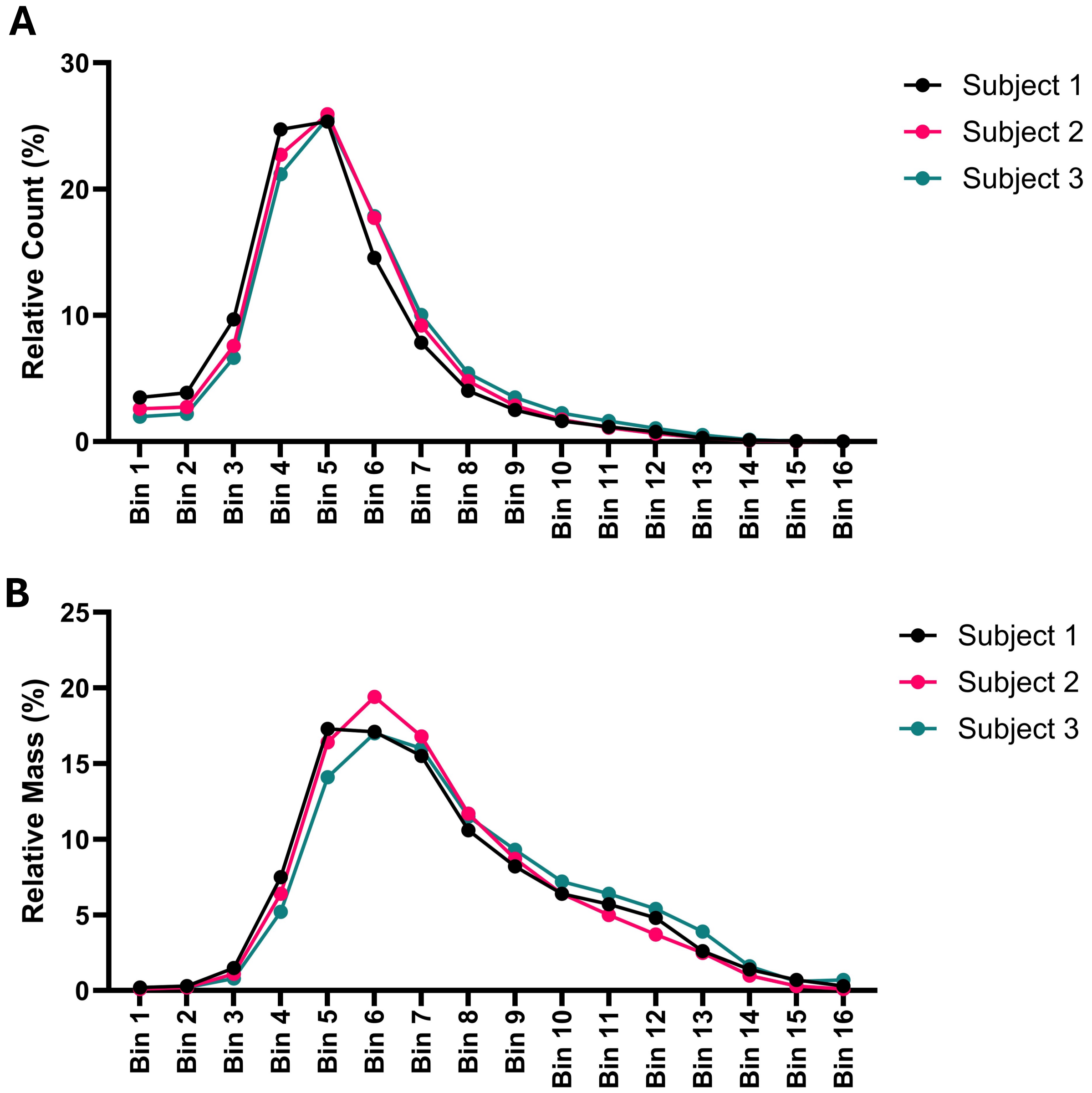

Figure S1. Particle-size distributions of PExA samples.
(A) Relative particle count and (B) relative particle mass across optical particle-counter size bins for the three matched PExA samples.
